# Dentine oxygen isotopes (*δ* ^18^O) as a proxy for odontocete distributions and movements

**DOI:** 10.1002/ece3.2238

**Published:** 2016-06-08

**Authors:** Cory J. D. Matthews, Fred J. Longstaffe, Steven H. Ferguson

**Affiliations:** ^1^ Fisheries and Oceans Canada 501 University Crescent Winnipeg Manitoba Canada; ^2^ Department of Earth Sciences The University of Western Ontario London Ontario Canada

**Keywords:** Carbonate, cetacean, distribution, hydroxyapatite, isoscape, marine mammal, oxygen isotopes, phosphate, teeth

## Abstract

Spatial variation in marine oxygen isotope ratios (*δ* ^18^O) resulting from differential evaporation rates and precipitation inputs is potentially useful for characterizing marine mammal distributions and tracking movements across *δ* ^18^O gradients. Dentine hydroxyapatite contains carbonate and phosphate that precipitate in oxygen isotopic equilibrium with body water, which in odontocetes closely tracks the isotopic composition of ambient water. To test whether dentine oxygen isotope composition reliably records that of ambient water and can therefore serve as a proxy for odontocete distribution and movement patterns, we measured *δ* ^18^O values of dentine structural carbonate (*δ* ^18^ O_SC_) and phosphate (*δ* ^18^ O_P_) of seven odontocete species (*n* = 55 individuals) from regional marine water bodies spanning a surface water *δ* ^18^O range of several per mil. Mean dentine *δ* ^18^ O_SC_ (range +21.2 to +25.5‰ VSMOW) and *δ* ^18^ O_P_ (+16.7 to +20.3‰) values were strongly correlated with marine surface water *δ* ^18^O values, with lower dentine *δ* ^18^ O_SC_ and *δ* ^18^ O_P_ values in high‐latitude regions (Arctic and Eastern North Pacific) and higher values in the Gulf of California, Gulf of Mexico, and Mediterranean Sea. Correlations between dentine *δ* ^18^ O_SC_ and *δ* ^18^ O_P_ values with marine surface water *δ* ^18^O values indicate that sequential *δ* ^18^O measurements along dentine, which grows incrementally and archives intra‐ and interannual isotopic composition over the lifetime of the animal, would be useful for characterizing residency within and movements among water bodies with strong *δ* ^18^O gradients, particularly between polar and lower latitudes, or between oceans and marginal basins.

## Introduction

Toothed whales (suborder Odontoceti) are widely distributed from tropical to polar regions, occupying coastal, shelf, and oceanic habitats (Forcada 2002). Despite the lack of apparent barriers to movement, many odontocetes display distinct structuring across their distributions, such as between coastal and offshore (Perrin 1984) or migratory and resident (Drouot et al. 2004) populations. Individuals of different sex, age, and reproductive status also have specific biological requirements that can lead to pronounced spatial segregation within populations (e.g., Rice 1989; Loseto et al. 2006). Temporal shifts in odontocete distributions generally reflect seasonal changes in habitat or prey distributions (e.g., Similä et al. 1996; Forney and Wade 2006), while long‐distance movements include dispersal from natal areas (Rice 1989) and migrations for breeding and purported physiological purposes (e.g., Durban and Pitman 2011).

Defining residency and movement patterns is important for understanding the ecological (e.g., prey and competitor distributions), demographic (e.g., age class and reproductive status), and habitat‐related factors that shape species distributions (Forcada 2002). However, considerable knowledge gaps concerning general distribution patterns persist for many odontocetes, especially oceanic species that maintain vast home ranges (e.g., MacLeod et al. 2006). Stable isotope analysis has become a popular approach for inferring animal distributions from the isotopic composition of their tissues (Hobson 1999). Regional variation in underlying biogeochemical processes leads to geographic patterns in stable isotope concentrations termed isoscapes (Graham et al. 2010; McMahon et al. 2013). Animal tissues take on these regional isotope characteristics via food and water, with some degree of predictable modification from baseline values (e.g., McCutchan et al. 2003; Caut et al. 2009), thereby becoming an intrinsic marker of distribution and movements across isotopically distinct regions of a species' range (e.g., Mendes et al. 2007a,b).

Strong latitudinal gradients in stable carbon and nitrogen isotope concentrations in the marine environment (Graham et al. 2010; McMahon et al. 2013) have provided spatial context for inferring large‐scale distribution patterns of marine mammals from tissue *δ* ^13^C and *δ* ^15^N values (e.g., Mendes et al. 2007a,b). Although more homogenous on a global scale than *δ* ^13^C and *δ* ^15^N values, oxygen isotope fractionation caused by evaporation and condensation of water vapor during atmospheric transport (Gat 1996) leads to marine surface water *δ* ^18^O variation of several per mil or greater between low and high latitudes, as well as between enclosed basins and adjacent oceans (LeGrande and Schmidt 2006; McMahon et al. 2013). Low‐latitude waters with high net evaporation rates are typically ^18^O‐enriched relative to higher latitudes, resulting in latitudinal marine *δ* ^18^O gradients of several per mil in both the Northern and Southern Hemispheres (LeGrande and Schmidt 2006). Such ^18^O enrichment is also typical of marginal seas with high net evaporation rates and limited exchange with the adjacent ocean, such as the Mediterranean and Red Seas, while high‐latitude water bodies with low net evaporation and ^18^O‐depleted precipitation, such as Hudson Bay and the Baltic Sea, have the lowest global marine surface water *δ* ^18^O values (LeGrande and Schmidt 2006; McMahon et al. 2013).

Biogenic apatite, the mineral component of bones and teeth, contains structural carbonate and phosphate that precipitate in oxygen isotopic equilibrium with body water, offset by temperature‐dependent fractionation that is held constant in homeothermic mammals (Longinelli 1984; Luz et al. 1984). The oxygen isotope composition of odontocete body water closely tracks that of ambient seawater because the dominant oxygen fluxes, ingested water and transcutaneous water exchange (Hui 1981; Andersen and Nielsen 1983), do not strongly fractionate oxygen (Kohn 1996). Accordingly, Yoshida and Miyazaki (1991) observed correlations between the oxygen isotope composition of bone phosphate of freshwater and marine cetaceans and ambient water. Oxygen isotopes in bone and enamel have since been used to differentiate between marine and freshwater habitats of ancient and extant marine mammals (Thewissen et al. 1996; Clementz and Koch 2001; Clementz et al. 2006), but studies of distribution and movements across marine *δ* ^18^O gradients have been limited (e.g., Killingley 1980; Borrell et al. 2013; Zenteno et al. 2013; Vighi et al. 2014).

Isotopic analysis of dentine, which grows continuously and retains its isotopic composition indefinitely (Bloom and Fawcett 1975), has recently proven useful for reconstructing individual marine mammal distributions and movements from *δ* ^13^C and *δ* ^15^N values of isolated collagen (Mendes et al. 2007a,b; Martin et al. 2011; Riofrío‐Lazo et al. 2012; Matthews and Ferguson 2014). To assess the applicability of *δ* ^18^O analysis of dentine hydroxyapatite (Ca_10_[PO_4_,CO_3_]_6_[OH,CO_3_]_2_) in this context, we measured *δ* ^18^O values of structural carbonate and phosphate (*δ* ^18^O_SC_ and *δ* ^18^O_P_) in dentine of odontocete species from marine water bodies ranging in surface water *δ* ^18^O values (LeGrande and Schmidt 2006). Our goals were to determine whether (1) dentine *δ* ^18^O_SC_ and *δ* ^18^O_P_ values reflect spatial differences in ambient seawater *δ* ^18^O values, and (2) *δ* ^18^O differences among specimens are sufficiently distinct to serve as an intrinsic marker of residency within and movements across regional marine *δ* ^18^O gradients.

## Methods

### Specimen collection and dentine sampling

**Figure 1:**
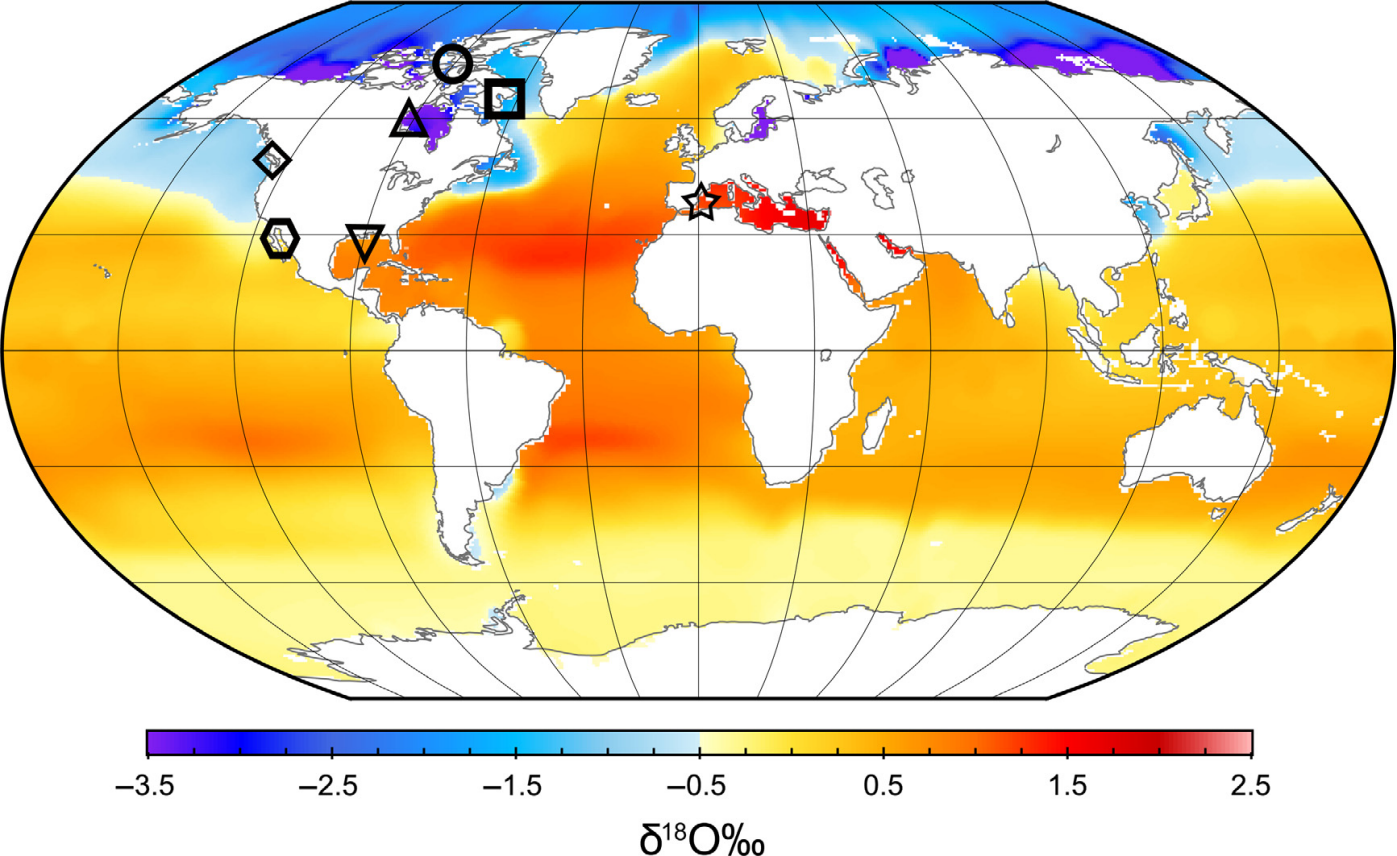
Teeth were collected from seven cetacean species distributed across a range of marine surface water *δ* ^18^O values: Eastern High Arctic‐Baffin Bay (EHA‐BB) belugas (circle); Cumberland Sound (CS) belugas (square); Western Hudson Bay (WHB) belugas (upright triangle), Eastern North Pacific (ENP) killer whales and harbour porpoises (diamond), Gulf of California (GOC) common bottlenose dolphins (hexagon), Gulf of Mexico (GOM) common bottlenose dolphins and Atlantic spotted dolphins (inverted triangle), and Mediterranean Sea (MED) short‐beaked common dolphin and a striped dolphin (star). Global gridded oxygen isotope data were made available through the Global Seawater Oxygen‐18 Database (Schmidt et al. 1999).

Teeth (*n* = 55 individuals) of seven odontocete species from marine water bodies spanning an approximate 4‰ range in surface water *δ* ^18^O values were acquired from government, museum, and private collections (Fig. 1; Table 1). Beluga (*Delphinapterus leucas*) teeth were collected from three eastern Canadian Arctic populations (Western Hudson Bay, *n* = 7; Cumberland Sound, *n* = 8; and Eastern High Arctic‐Baffin Bay, *n* = 9), representing the low end of the global range of marine surface water *δ* ^18^O values (Fig. 1; Table 1). Killer whale (*Orcinus orca*,* n* = 2) and harbour porpoise (*Phocoena phocoena*,* n* = 8) teeth were collected from coastal British Columbia, where surface water *δ* ^18^O values average slightly higher than in the Arctic (Fig. 1; Table 1). Teeth collected from common bottlenose dolphins (*Tursiops truncatus*,* n* = 5) from the Gulf of California, common bottlenose dolphins (*n* = 8) and Atlantic spotted dolphins (*Stenella frontalis*,* n* = 3) from the Gulf of Mexico, and short‐beaked common dolphins (*Delphinus delphis*,* n* = 4) and a striped dolphin (*Stenella coeruleoalba*) from the Mediterranean Sea represented marine water bodies with high surface water *δ* ^18^O values (Fig. 1; Table 1). Specimens were collected over 1965–2008 and stored dry, except for beluga teeth, which were frozen in jaws until they were excised for dentine sampling.

**Table 1:** Marine surface water *δ* ^18^O (‰) values of regional water bodies represented by odontocete specimens. Data were downloaded from the Global Seawater Oxygen‐18 Database, version 1.21 (Schmidt et al. 1999)

Location	Depth (m)	Years sampled	*δ* ^18^O (‰) mean ± SD	*δ* ^18^O (‰) range	References
Canadian Arctic – Eastern High Arctic‐Baffin Bay (EHA‐BB)	0–250	1974–1980	−1.58 ± 0.57 (*n* = 535)	−3.31 to +0.11	Tan and Strain (1980)
Canadian Arctic – Cumberland Sound (CS)	0–244	1977	−1.74 ± 0.40 (*n* = 19)	−2.33 to −0.88	Tan and Strain (1980)
Canadian Arctic – Western Hudson Bay (WHB)	0–242	1982	−2.57 ± 0.65 (*n* = 180)	−4.18 to −1.42	Tan and Strain (1996), Schriber et al. (1974), Bédard et al. (1981)
Eastern North Pacific (ENP)	0–1	1950–1973	−0.73 ± 0.27 (*n* = 9)	−1.12 to −0.28	Craig and Gordon (1965), Epstein and Mayeda (1953), Ostlund et al. (1987)
Gulf of California (GOC)	0–50	not measured^a^	+0.20 ± 0.20^a^	not measured^a^	LeGrande and Schmidt (2006)
Gulf of Mexico (GOM)	74–220	1983–1984	+1.00 ± 0.19 (*n* = 8)	+0.7 to +1.2	Grossman and Ku (1986)
Mediterranean Sea (MED)	0–250	1986–1990	+1.31 ± 0.20 (*n* = 97)	+0.7 to +1.67	Pierre et al. (1986), Pierre (1999)

a Estimated from regional *δ* ^18^O to salinity relationships. SD of 0.20‰ is used for consistency with the Gulf of Mexico and Mediterranean Sea.

Teeth were sectioned longitudinally along the midline using a water‐cooled diamond‐coated saw blade. Dentine was sampled from one of the two sections using a micromill (New Wave Research, Freemont, California) fitted with a 1‐mm‐diameter carbide drill bit. Each sample comprised all dentine annuli and therefore represents whole‐tooth deposition. The small size of harbour porpoise teeth required drilling of both sections to acquire sufficient material for analysis.

### Oxygen isotope analysis of dentine

All stable isotope analyses were carried out in the Laboratory for Stable Isotope Science (LSIS) at the University of Western Ontario and are reported in *δ*‐notation relative to Vienna Standard Mean Ocean Water (VSMOW) using two‐point calibrations following Coplen (1996) and Coplen et al. (2006). Bioapatite is commonly treated to remove organic matter and secondary carbonate prior to *δ* ^18^O analysis of structural carbonate. However, numerous studies (e.g., Snoeck and Pellegrini 2015; Pellegrini and Snoeck 2016) have shown that pretreatment can have unintended and inconsistent consequences for isotopic composition of structural carbonate. We therefore performed test comparisons between untreated dentine samples and those reacted with 2% sodium hypochlorite and 0.1 M acetic acid to remove organics and secondary carbonates. We found treated samples had lower *δ* ^18^O_SC_ values that were consistent with isotopic exchange between poorly crystallized structural carbonate and water in the hypochlorite and acetic acid solutions during the bleaching and acidification steps. We therefore proceeded with analysis of untreated dentine, which was finely powdered, placed in a reaction vial (~0.8–1.0 mg), dried overnight at 80°C, and then septa‐sealed and capped in preparation for isotopic analysis of structural carbonate. A Micromass MultiPrep automated sampling device was used to evacuate the vial and then introduce orthophosphoric acid to generate carbon dioxide gas (CO_2_) by reaction with the sample at 90°C for 20 min. The evolved CO_2_ was then cryogenically scrubbed of contaminants and automatically transferred to a VG Optima isotope‐ratio mass spectrometer (IRMS) for isotopic analysis in dual‐inlet mode.

Dentine *δ* ^18^O_SC_ values were calibrated relative to VSMOW using accepted values for NBS‐19 (+28.65‰) and NBS‐18 (+7.20‰), with a precision (SD) of ±0.08‰ (*n* = 16) and 0.11‰ (*n* = 8), respectively. Accuracy and precision (SD) were assessed using internal laboratory reference materials not included in the calibration curve: WS‐1 calcite (*δ* ^18^O measured = +26.27 ± 0.13‰, *n* = 7; accepted = +26.23‰) and Suprapur (*δ* ^18^O measured = +13.29 ± 0.10‰, *n* = 6; accepted = +13.30‰). The average *δ* ^18^O difference between duplicate analyses of samples was ± 0.16‰ (*n* = 7).

Samples were prepared for *δ* ^18^O_P_ analysis by dissolving ~25–35 mg of each powdered dentine sample in 3 M acetic acid. Silver phosphate (Ag_3_PO_4_) was then precipitated through several chemical intermediaries (lead phosphate, lead sulfate) following the ammonia volatilization method (Firsching 1961; Stuart‐Williams and Schwarcz 1995). Approximately 0.2 mg of powdered Ag_3_PO_4_ was then loaded into silver capsules and introduced into a Thermo Scientific High Temperature Conversion Elemental Analyzer (TC/EA) using a zero blank autosampler. Following reaction at 1350°C for a few seconds with the TC/EA glassy carbon tube, the resulting carbon monoxide (CO) gas was passed through a heated (120°C) homemade GC column packed with a 5 Å molecular sieve to eliminate impurities such as water vapor. The CO was then swept using helium gas in continuous flow mode to a Thermo Scientific Delta^PLUS^XL IRMS (Darmstadt, Germany) for isotopic analysis. Limited sample amounts prevented *δ* ^18^O_P_ analysis of teeth from harbour porpoises and Mediterranean Sea dolphins.

Dentine *δ* ^18^O_P_ values were calibrated relative to VSMOW using accepted values of IAEA‐CH‐6 (+36.40‰; Flanagan and Farquhar 2014) and Aldrich Silver Phosphate – 98%, Batch 03610EH (+11.2‰; Webb et al. 2014), with a precision (SD) of ± 0.26‰ (*n* = 7) and 0.28‰ (*n* = 12), respectively. The average (±SD) *δ* ^18^O value of five replicate analyses of phosphate extracted from NBS 120c (accepted value = +21.7‰; Lécuyer et al. 2013) was +21.17 ± 0.14‰. The average difference between replicate analyses of samples was ± 0.27‰ (*n* = 9), including two method duplicates in which a separate aliquot of silver phosphate was prepared from original dentine.

### Data analysis

Correlations between mean dentine *δ* ^18^O_SC_ and *δ* ^18^O_P_ values (averaged by species within each water body) and mean marine surface water *δ* ^18^O values were determined using linear regression. Marine surface water *δ* ^18^O measurements restricted to the upper 250 m of each water body were downloaded from the Global Seawater Oxygen‐18 Database, version 1.21 (Schmidt et al. 1999). Surface marine water *δ* ^18^O measurements (1950s to 2000s) generally overlapped the period of tooth collection/dentine deposition, but were limited to just one or several years in some regions (Table 1). We assume sparsely collected marine water *δ* ^18^O data are representative of the long‐term mean (LeGrande and Schmidt 2006), and that dentine and marine water *δ* ^18^O values are comparable in cases when dentine deposition and water sampling have minimal temporal overlap. Surface marine water *δ* ^18^O measurements were unavailable for the Gulf of California, so a model‐derived estimate based on salinity (LeGrande and Schmidt 2006) was used instead.

Differences in mean *δ* ^18^O_SC_ and *δ* ^18^O_P_ among species grouped by water body were assessed using one‐way ANOVA, with significant differences between groups determined using Tukey honestly significant difference (HSD) post hoc pairwise comparisons. Examination of residual vs. fitted values indicated the homogeneity of variance assumption was violated for *δ* ^18^O_SC_ values, and so a one‐way test with a Welch correction for unequal variances was performed instead (Welch 1951), followed by Games–Howell post hoc pairwise comparisons. Analyses were conducted using base functions and the “userfriendlyscience” package (Peters 2015) available for R software (R Core Team 2012).

## Results

**Figure 2:**
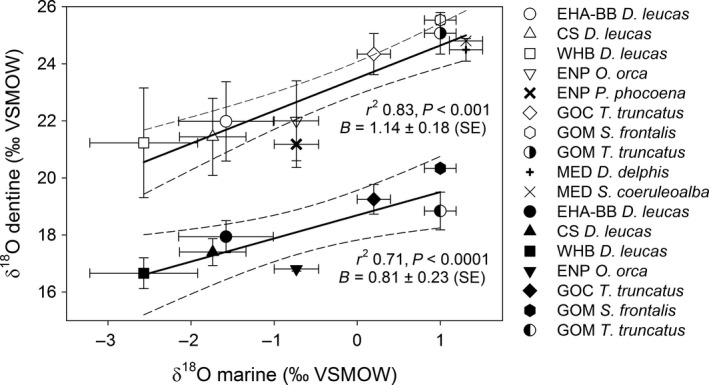
Mean dentine *δ* ^18^ O_SC_ (hollow symbols) and *δ* ^18^ O_P_ (solid symbols) values were positively correlated with mean surface marine water *δ* ^18^O values of the regional water bodies from which the specimens originated (B = slope): Eastern High Arctic‐Baffin Bay (EHA‐BB), Cumberland Sound (CS), Western Hudson Bay (WHB), Eastern North Pacific (ENP), Gulf of California (GOC), Gulf of Mexico (GOM), and the Mediterranean Sea (MED). Surface marine water *δ* ^18^O values were downloaded from the Global Seawater Oxygen‐18 Database (Schmidt et al. 1999) and are presented in Table 1. 95% confidence bands shown by dashed lines.

Mean dentine *δ* ^18^O_SC_ values grouped by species within water body ranged from +21.2‰ (harbour porpoises from the Eastern North Pacific) to +25.5‰ (Atlantic spotted dolphins from the Gulf of Mexico; Table 2). Mean dentine *δ* ^18^O_SC_ values were significantly correlated with mean surface water *δ* ^18^O values (*R* ^2^ 0.84, *F*_1,8_ = 41.90, *P *<* *0.0001), with slope and intercept estimates of 1.13 ± 0.18 (SE) and 23.53 ± 0.24, respectively (Fig. 2). Eastern North Pacific harbour porpoises and killer whales had lower *δ* ^18^O_SC_ values than predicted from surface marine water *δ* ^18^O values (Fig. 2).

**Table 2:** Mean (±SD) oxygen isotope compositions (‰ VSMOW) of dentine carbonate (*δ* ^18^O_SC_) and phosphate (*δ* ^18^O_P_) in teeth of cetaceans from marine water bodies that span a range of surface water *δ* ^18^O values

Location	Species	Common name	*δ* ^18^O_SC_ (‰)	*δ* ^18^O_P_ (‰)
Canadian Arctic	*Delphinapterus leucas* (Eastern High Arctic‐Baffin Bay population)	Beluga	+21.98 ± 1.39 (*n* = 9)	+17.94 ± 0.56 (*n* = 8)
	*D. leucas* (Cumberland Sound population)	Beluga	+21.44 ± 1.35 (*n* = 8)	+17.40 ± 0.47 (*n* = 8)
	*D. leucas* (Western Hudson Bay population)	Beluga	+21.23 ± 1.92 (*n* = 7)	+16.66 ± 0.54 (*n* = 7)
Eastern North Pacific	*Orcinus orca*	killer whale	+22.00 ± 1.40 (*n* = 2)	+16.81 ± 0.08 (*n* = 2)
	*Phocoena phocoena*	harbour porpoise	+21.18 ± 0.81 (*n* = 8)	Not available
Gulf of California	*Tursiops truncatus*	common bottlenose dolphin	+24.34 ± 0.72 (*n* = 5)	+19.25 ± 0.52 (*n* = 5)
Gulf of Mexico	*Stenella frontalis*	Atlantic spotted dolphin	+25.53 ± 0.16 (*n* = 3)	+20.34 (*n* = 1)
	*T. truncatus*	common bottlenose dolphin	+25.07 ± 0.73 (*n* = 8)	+18.84 ± 0.66 (*n* = 7)
Mediterranean Sea	*Delphinus delphis*	short‐beaked common dolphin	+24.49 ± 0.40 (*n* = 4)	Not available
	*Stenella coeruleoalba*	striped dolphin	+24.8 (*n* = 1)	Not available

Mean dentine *δ* ^18^O_P_ values ranged from +16.7‰ in Hudson Bay belugas to +20.3‰ in Atlantic spotted dolphins from the Gulf of Mexico (Table 2). Mean dentine *δ* ^18^O_P_ and surface water *δ* ^18^O values were significantly correlated (*R* ^2^ 0.75, *F*_1,5_ = 15.23, *P *<* *0.0001), with slope and intercept estimates of 0.81 ± 0.21 (SE) and 18.73 ± 0.30, respectively (Fig. 2). As with *δ* ^18^O_SC_ values, mean *δ* ^18^O_P_ values of Eastern North Pacific killer whales fell below the 95% confidence bands of the regression (Fig. 2).

Mean dentine *δ* ^18^O_SC_ values differed significantly among specimens from different water bodies (ANOVA, *F*_6,48_ = 18.46, *P *<* *0.001). Mean *δ* ^18^O_SC_ values of eastern Canadian Arctic and Eastern North Pacific specimens were statistically indistinguishable (*P *>* *0.8), but differed from those from the Gulf of California, Gulf of Mexico, and Mediterranean Sea (*P *<* *0.05). The *δ* ^18^O_SC_ values of specimens from the latter three water bodies were statistically indistinguishable (*P *>* *0.2).

Significant differences among mean dentine *δ* ^18^O_P_ values (ANOVA, *F*_5,32_ = 17.19, *P *<* *0.001) occurred among the same groups as *δ* ^18^O_SC_ values (*P *<* *0.001). Additionally, mean *δ* ^18^O_P_ values differed significantly between Hudson Bay and EHA‐BB belugas (*P *<* *0.01).

## Discussion

### Variation in dentine δ^18^O_SC_ and δ^18^O_P_ values

As expected, much of the variation in specimen dentine *δ* ^18^O_SC_ and *δ* ^18^O_P_ values was explained by geographic variation in the stable oxygen isotope composition of ambient seawater, which is consistent with previous studies of bone phosphate *δ* ^18^O variation in freshwater and marine cetaceans (Yoshida and Miyazaki 1991) and turtles (Barrick et al. 1999; Coulson et al. 2008). Our slope and intercept estimates (±SE) for dentine *δ* ^18^O_P_ values are similar to Yoshida and Miyazaki's (1991) regression of cetacean bone phosphate *δ* ^18^O values against ambient water (0.773 and 17.8, respectively), despite their correlation being driven largely by the inclusion of river dolphins from habitats with relatively low *δ* ^18^O values (bone *δ* ^18^O_SC_ values were not measured in their study).

Ambient temperature variation was an unavoidable consequence of our selection of odontocete specimens representing a latitudinal gradient in marine *δ* ^18^O values. Although bioapatite precipitates at constant body temperature in mammals, ambient temperature gradients could potentially influence the oxygen isotope composition of teeth that are in contact with surrounding water. Barrick et al. (1992) found phosphate *δ* ^18^O values of rostral bones and teeth of cetaceans were 0.3‰ greater than those of vertebrae from the same animals, which they attributed to a 1–1.5°C heat loss from water‐flushed jaws relative to the body core. Presumably, any cooling influence leading to higher dentine *δ* ^18^O values would be relatively greater for cold‐water species, such as belugas, or smaller species whose jaws may be more exposed, such as the harbour porpoise. However, neither belugas nor harbour porpoises had higher *δ* ^18^O values than predicted from surface water *δ* ^18^O values, suggesting any influence of ambient water temperature on oxygen isotope fractionation during dentine precipitation is negligible.

Our selection of different odontocete species from a broad range of marine *δ* ^18^O values also introduced interspecies variability as a potential factor in our analysis (e.g., Barrick et al. 1992 attributed higher than expected bone *δ* ^18^O_P_ values in sperm whales, *Physeter macrocephalus*, to their lower body temperature relative to other cetaceans). The only consistent offsets from expected values in our regressions were harbour porpoises and killer whales from the Eastern North Pacific, whose dentine *δ* ^18^O values were lower than predicted from ambient surface water values. While the large difference in body size between these two species makes a common physiological explanation unlikely, inaccurate seawater *δ* ^18^O values due to poor spatial and temporal coverage of measurements in the region (Schmidt et al. 1999), along with coastal habitat preferences of the two species, are a plausible explanation for the observed discrepancy. ^18^O‐depleted riverine inputs typically lower the *δ* ^18^O values of coastal waters (McMahon et al. 2013), and the coastal habitats of Pacific harbour porpoises, which occur in inlets, bays, and estuaries at depths typically less than 100 m (Baird 2003), are characterized by average *δ* ^18^O values (~−5‰; Gillikin et al. 2005) well below those used for the Eastern North Pacific in our regression analysis (Table 1). Killer whales also occupy a coastal distribution off British Columbia and Washington during spring to fall, although they range widely from Alaska to California during the winter months (Forney and Wade 2006). The vaguely defined seasonal ranges of killer whales in the Eastern North Pacific may have resulted in a mismatch between regional marine *δ* ^18^O values used in the regression and their actual distribution.

Differences in *δ* ^18^O values between the two Gulf of Mexico dolphin species (which were not significant [*P *=* *0.08], likely due to small sample sizes; see Clementz and Koch 2001) may also reflect different habitat preferences of the two species. Spotted dolphins in the Gulf of Mexico, which prefer mid‐shelf habitats ranging from 20 to 180 m depth, had higher *δ* ^18^O values than common bottlenose dolphins, which commonly occur in shelf waters <20 m (Griffin and Griffin 2003). Habitat partitioning along these lines would be expected to produce the observed results based on a slight nearshore–offshore gradient in surface water *δ* ^18^O values in the Gulf of Mexico (LeGrande and Schmidt 2006; McMahon et al. 2013).

Similar habitat partitioning, combined with inherent variability in Arctic seawater *δ* ^18^O values, may explain the greater variation in beluga dentine *δ* ^18^O_SC_ values relative to the other species. Belugas segregate spatially by sex, age class, and reproductive status during the open water season, with females and calves occurring in coastal estuaries to which they are philopatric, and adult males more frequently found in deeper, offshore waters (Caron and Smith 1990; Loseto et al. 2006; Colbeck et al. 2013). Estuarine waters reflect a mix of marine and freshwater inputs with lower surface water *δ* ^18^O values than offshore waters inhabited by adult males, introducing potential variation between females and males, while philopatry to a given estuary with distinct riverine flow rates and/or *δ* ^18^O values could also introduce variation in dentine *δ* ^18^O values among females. Further, Arctic seawater *δ* ^18^O values are more spatially and temporally variable than ice‐free waters due to seasonal freezing and melting of sea ice (Tan and Strain 1980, 1996; Bédard et al. 1981). Sea ice weakly preferentially incorporates ^18^O (Tan and Fraser 1976), so sea ice meltwater is slightly ^18^O‐enriched relative to marine water. The inherent variability in Arctic surface water *δ* ^18^O values reflecting inputs of ^18^O‐depleted meteoric water and ^18^O‐enriched meltwater, coupled with habitat segregation during summer months when the bulk of dentine deposition is thought to occur (Klevezal 1996), could be a driver of the higher variation observed in beluga dentine *δ* ^18^O_SC_ values. Unfortunately, sample sizes were too small to examine dentine *δ* ^18^O differences between females and males, or among juvenile and adult animals, which would extend from this hypothesis.

Variation in beluga *δ* ^18^O_P_ measurements, however, was considerably lower than for *δ* ^18^O_SC_ values and was comparable to that of the other groups, suggesting habitat is not the primary cause of high *δ* ^18^O_SC_ variation in belugas (since both carbonates and phosphates precipitate in isotopic equilibrium from the same oxygen pool, they would presumably vary in a similar manner; Iacumin et al. 1996). Further, the ~5‰ difference between dentine *δ* ^18^O_SC_ and *δ* ^18^O_P_ values (Δ^18^O_SC‐P_) is less than that measured in bone and enamel bioapatite of terrestrial mammals (~8–9‰; Bryant et al. 1996; Iacumin et al. 1996; Martin et al. 2008). While our *δ* ^18^O_P_ values are similar to those previously measured in cetacean bone phosphate (~+17 to +19‰; Yoshida and Miyazaki 1991; Barrick et al. 1992), our *δ* ^18^O_SC_ measurements are lower than those previously measured in cetacean enamel and dentine (+27.8 to +28.5‰, Clementz and Koch 2001; +29.8 to +29.9‰, Borrell et al. 2013), as well as bone (+29.5‰, Vighi et al. 2014).

A possible explanation for the lower than expected *δ* ^18^O_SC_ values is that isotopic analysis of bioapatite structural carbonate without first removing organic matter, as was done in this study, could have caused the bioapatite crystals to remain armored (e.g., Munro et al. 2008) from interaction with the orthophosphoric acid, thus preventing complete reaction. This is unlikely, however, because CO_2_ yields are within the normal range for structural carbonate, and there is no correlation between *δ* ^18^O_SC_ and CO_2_ yield. A second possibility is that oxygen released from collagen during the dentine‐orthophosphoric acid reaction exchanged with carbon dioxide released from structural carbonate. Studies of terrestrial mammals indicate that collagen is ^18^O‐depleted relative to coexisting structural carbonate (Δ^18^O_SC‐collagen_ = ~13–18‰; e.g., Crowley 2014). However, it is unlikely that oxygen was released from collagen during the dentine‐orthophosphoric reaction at 90°C, as tests of reaction of pure collagen we conducted under these conditions produced no oxygen or oxygen‐bearing species convertible to CO_2_. A third explanation, which we currently favor, is isotopic exchange between CO_2_ produced during the dentine‐orthophosphoric acid and residual water associated with dentine collagen. Our drying procedure (80°C) may have been insufficient to remove all water associated with collagen. Assuming that this water has a *δ* ^18^O value similar to odontocete body water (~0‰), its exchange with CO_2_ released from structural carbonate could drive down *δ* ^18^Osc values, while retaining the strong linear correlation observed between dentine *δ* ^18^O_SC_ and ambient seawater *δ* ^18^O values.

### δ^18^O values as a proxy for odontocete distribution and movements

The strong correlations between dentine *δ* ^18^O_SC_ and *δ* ^18^O_P_ values and ambient seawater *δ* ^18^O values indicate dentine *δ* ^18^O values can serve as a suitable proxy for odontocete distributions across the marine *δ* ^18^O isoscape, particularly across latitudinal *δ* ^18^O gradients or between marginal seas and adjacent oceans.

Cross‐sectional studies of populations using whole‐tooth dentine *δ* ^18^O measurements, which integrate long‐term isotopic deposition, would be suitable for broad investigations of spatial structuring across a species' range (see Zenteno et al. 2013 and Vighi et al. 2014; who used *δ* ^18^O values of sea lion [*Otaria byronia*] and southern right whale [*Eubalaena australis*] bone, respectively, to reveal dispersal patterns and population structuring). Significant differences in mean dentine *δ* ^18^O_P_ values between Western Hudson Bay and Eastern High Arctic‐Baffin Bay belugas are consistent with genetics and satellite telemetry data that show no distributional overlap occur between these populations (Richard et al. 1990; Brennin et al. 1997). However, the lack of differences between Western Hudson Bay and Cumberland Sound belugas, and between CS and EHA‐BB belugas, even though these populations are also geographically distinct, suggests that either ambient surface water *δ* ^18^O values are not sufficiently distinct to distinguish distributional differences between them, or statistical power of our small sample sizes was insufficient to detect them.

Longitudinal oxygen isotope data acquired from within‐tooth dentine sampling, on the other hand, would be suitable for reconstructing individual movements, such as those by recently satellite‐tagged killer whales spanning pronounced latitudinal *δ* ^18^O gradients in both the Northern and Southern Hemispheres (Matthews et al. 2011; Durban and Pitman 2011), or by male sperm whales dispersing from natal areas in the tropics to high‐latitude foraging grounds (Rice 1989). Chronological *δ* ^18^O profiles from sequentially sampled dentine annuli could be used to determine, for example, whether individuals make repeated annual migrations, or to link dispersal and migrations with particular life‐history events (e.g., sexual maturation). Borrell et al. (2013) used ontogenetic *δ* ^18^O patterns across individual dentine annuli to show dissimilar migration patterns between sperm whales from Denmark and northwest Spain. Seasonal migration reconstructions would require micromilling of alternating light and dark annuli thought to be deposited seasonally (Klevezal 1996) or microspatial sampling within annuli (see Cerling and Sharp 1996; Sharp and Cerling 1998), and sufficiently long residency in each destination to be recorded in dentine.

Ecological interpretations of tissue *δ* ^13^C and *δ* ^15^N values can be complicated by the confounding influences of trophic and physiological factors, as well as baseline variation on multiple spatial and temporal scales (see Matthews and Ferguson 2014). In contrast, the oxygen isotope composition of freshwater and marine vertebrate tissues primarily reflects that of ambient water (Yoshida and Miyazaki 1991; Coulson et al. 2008; this study). We therefore suggest oxygen isotope analysis is an underused approach for inferring marine mammal distributions that could be employed along with more common isotope proxies, such as *δ* ^13^C and *δ* ^15^N analysis of dentine collagen, or in conjunction with telemetry and genetics studies of distribution and population structure.

## Conflict of Interest

None declared.
